# Upregulation of 15 Antisense Long Non-Coding RNAs in Osteosarcoma

**DOI:** 10.3390/genes12081132

**Published:** 2021-07-26

**Authors:** Emel Rothzerg, Xuan Dung Ho, Jiake Xu, David Wood, Aare Märtson, Sulev Kõks

**Affiliations:** 1 School of Biomedical Sciences, The University of Western Australia, Perth, WA 6009, Australia; emel.rothzerg@research.uwa.edu.au (E.R.); jiake.xu@uwa.edu.au (J.X.); david.wood@uwa.edu.au (D.W.); 2 Perron Institute for Neurological and Translational Science, QEII Medical Centre, Nedlands, WA 6009, Australia; 3 Department of Oncology, College of Medicine and Pharmacy, Hue University, Hue 53000, Vietnam; xuandung59@gmail.com; 4 Department of Traumatology and Orthopaedics, University of Tartu, Tartu University Hospital, 50411 Tartu, Estonia; aare.martson@kliinikum.ee; 5 Centre for Molecular Medicine and Innovative Therapeutics, Murdoch University, Murdoch, WA 6150, Australia

**Keywords:** osteosarcoma, sarcoma, non-coding RNA, antisense RNA, alternative splicing

## Abstract

The human genome encodes thousands of natural antisense long noncoding RNAs (lncRNAs); they play the essential role in regulation of gene expression at multiple levels, including replication, transcription and translation. Dysregulation of antisense lncRNAs plays indispensable roles in numerous biological progress, such as tumour progression, metastasis and resistance to therapeutic agents. To date, there have been several studies analysing antisense lncRNAs expression profiles in cancer, but not enough to highlight the complexity of the disease. In this study, we investigated the expression patterns of antisense lncRNAs from osteosarcoma and healthy bone samples (24 tumour-16 bone samples) using RNA sequencing. We identified 15 antisense lncRNAs (*RUSC1-AS1*, *TBX2-AS1*, *PTOV1-AS1*, *UBE2D3-AS1*, *ERCC8-AS1*, *ZMIZ1-AS1*, *RNF144A-AS1*, *RDH10-AS1*, *TRG-AS1*, *GSN-AS1*, *HMGA2-AS1*, *ZNF528-AS1*, *OTUD6B-AS1*, *COX10-AS1* and *SLC16A1-AS1*) that were upregulated in tumour samples compared to bone sample controls. Further, we performed real-time polymerase chain reaction (RT-qPCR) to validate the expressions of the antisense lncRNAs in 8 different osteosarcoma cell lines (SaOS-2, G-292, HOS, U2-OS, 143B, SJSA-1, MG-63, and MNNG/HOS) compared to hFOB (human osteoblast cell line). These differentially expressed IncRNAs can be considered biomarkers and potential therapeutic targets for osteosarcoma.

## 1. Introduction

Osteosarcoma (OS), also known as osteogenic sarcoma, is the most common primary malignant solid tumour of bone [1]. The peak incidence is in children and adolescents with a smaller second peak in incidence after the age of 65 years associated with Paget’s disease of bone [2]. OS commonly develops in the extremities of long bones such as the distal femur, proximal tibia, proximal humerus, and proximal femur [3]. It is an aggressive-invasion sarcoma type that frequently metastasizes to the lung and other bones in the body [4]. OS usually presents with pain, tenderness and swelling around the affected bone, and diagnosis is achieved by a combination of imaging and histology with the characteristic appearance of malignant cells forming osteoid [5]. Cytotoxic chemotherapy was introduced by Rosen in the 1970s and improved the prognosis from 20% to a 70% five-year survival rate with no further significant improvements in outcome since then [6]. Current treatments of OS include neo-adjuvant chemotherapy with drugs such as doxorubicin, methotrexate, and cisplatin with the aim of reducing tumour size as well as eradicating micro-metastases. Ablative surgery is then followed by further chemotherapy determined by the cell death rate observed in the surgical specimens [7,8]. Current OS therapeutic agents are limited to cytotoxic drugs interfering with transcription and DNA replication [6]. This is a reflection of our knowledge of the pathways involved in OS initiation and progression, which are insufficient to understand the underlying molecular mechanisms of the disease.

The sense strand of DNA provides the template for production of messenger RNA (mRNA) to be translated into proteins [9], but the Human Genome Project highlighted that only 1.5% of the human genome contains protein-coding genes. In addition, the Encyclopedia of DNA elements (ENCODE) and the Functional Annotation of the Mammalian Genome (FANTOM) have suggested that the majority of the genome is transcribed and produces a various amount of non-coding RNA species (ncRNAs) [10,11]. The ncRNAs, fittingly, are RNA molecules that do not encode proteins. They are generally classified based on their length, with an artificial cut off of 200 base pair (bp), small ncRNAs (sncRNAs) less than 200 bps, whereas long ncRNAs (lncRNAs) greater than 200 bps [12]. The lncRNAs frequently regulate epigenetic silencing through chromatin remodeling [13]. They also play critical role in regulating splicing, recruiting transcription factors, and controlling mRNA stability [14]. The natural antisense RNAs belong to the lncRNAs family and are transcribed from the opposite strand of a protein-coding gene [12]. They can stimulate, reduce or completely silence gene expression of the sense transcripts at multiple levels, play a functional role in physiological, and pathological processes, and may eventually lead to diseases [10,11,12,13,14,15].

**Figure 1:**
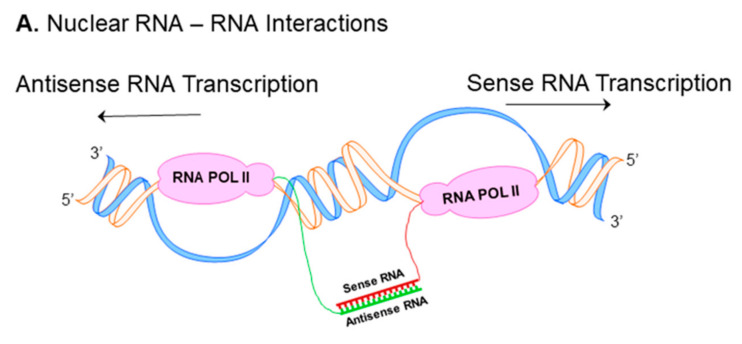

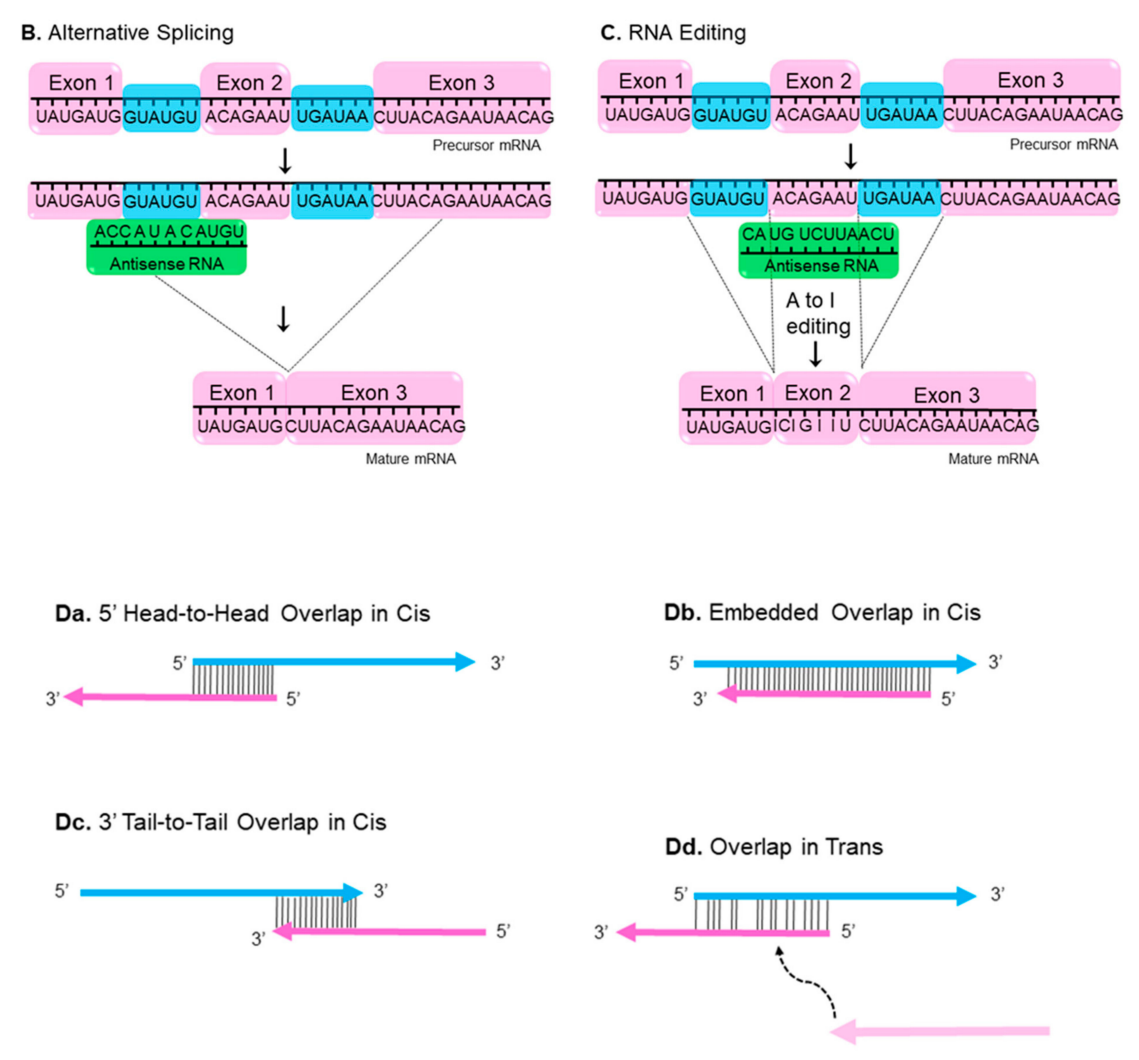
Nuclear and cytoplasmic sense–antisense RNA pairing. Diagram of nuclear RNA duplex formation (**A**) that further results in alternative splicing (**B**) or RNA editing (**C**). Classification of sense/antisense pairs (**D**). Sense genes represent in blue, whereas antisense transcripts are pink. The black lines between the genes indicates regions of overlap. There are different types of natural antisense transcripts overlapping: head-to-head overlap in *cis* (**Da**), embedded overlap in *cis* (**Db**), tail-to-tail overlap in *cis* (**Dc**), and overlap in *trans* (**Dd**).

Nuclear RNA duplex formation may occur locally immediately upon transcription, consequently resulting in inhibition of sense RNA processing (Figure 1A) [16]. The processing of RNA includes capping, polyadenylation, nuclear localization and mRNA transport, all these events may be affected by nuclear sense–antisense RNA duplex formation. Natural antisense transcripts (NATs) can cover donor (5′) and acceptor (3′) splice sites in the sense precursor mRNA transcript to modify alternative splicing patterns and develop mature RNA in different isoforms (Figure 1B) [16,17]. Another possible consequence of nuclear RNA duplex formation is RNA editing through the adenosine deaminases that act on the RNA (ADAR) enzyme responsible for binding to double stranded RNA and converting adenosine (A) to inosine (I) by hydrolytic deamination (an adenosine loses an amine group) (Figure 1C) [18,19].

NATs can be subdivided into two categories: *cis*-NATs and *trans*-NATs. *cis*-NATs are antisense RNAs transcribed from the same genomic *locus*. Consequently, in the section of the overlap, sense and antisense transcripts share complete complementarities such as head-to-head overlap in *cis* (Figure 1Da), embedded overlap in *cis* (Figure 1Db), and tail-to-tail overlap in *cis* (Figure 1Dc) [20]. On the other hand, *trans*-NATs are antisense RNAs transcribed from a different genomic region of their paired sense transcript, displaying partially complementarities such as overlap in *trans* (Figure 1Dd) [20,21,22].

Considering the growing evidence of the antisense lncRNAs in cellular process and their involvement in various disease types including cancer we investigated their expression pattern in OS using RNA sequencing (RNA-seq).

## 2. Materials and Methods

### 2.1. Sample Description

The study has been approved by the Ethics Review Committee of the University of Western Australia and Sir Charles Gairdner Hospital (2019/RA/4/20/5211). The patient informed consent forms were signed and dated by the participants and patient representatives before the limb sparing or amputation surgery.

Twenty-one Australian patients underwent the surgery to remove malignant tissue and the diagnosis of OS was confirmed by a specialist sarcoma pathologist. Cancerous and normal bone formalin-fixed paraffin-embedded (FFPE) tissue samples were collected from PathWest (QEII Medical, Nedlands, WA, Australia).

### 2.2. Total RNA Extraction and Sequencing

Total RNA was extracted from recently cut 5 sections of ≤ 20 μm thick FFPE samples using the FFPE RNA purification kit (Norgen Biotek, Thorold, ON, Canada), following to the manufacturer’s protocol. Extracted RNA was completely dissolved in RNase-free water and stored at –80 °C. The quality of total RNA was measured using Agilent 2100 Bioanalyzer and the RNA 6000 Nano Kit (Agilent Technologies Inc., Santa Clara, CA, USA).

The RNA samples were processed using the Takara SMARTer V2 Total RNA Mammalian Pico Input protocol using 2 ng of Total RNA input as per manufacturer’s instructions (Takara Bio Inc., Mountain View, CA, USA). The sequencing was completed using an Illumina NovaSeq 6000 and an S4-300 cycle lane (150PE) with v1.5 sequencing chemistry [23]. The FastQC (version 0.11.9) was used to determine the quality score distribution of the sequencing reads. The low-quality reads, Phred score ≤ 20, were trimmed out using Trimmomatic (version 0.39) [24]. Trimmed reads were mapped to the human genome hg38 (GRCH38) reference using STAR (version 2.7.7a) [25].

### 2.3. Data and Statistical Analyses

Differential gene expression and statistical data analyses were performed through DESeq2 package for R [26]. DESeq2 is a Bioconductor package specifically designed to detect differential expressed genes between individual samples. Differential gene expression levels between tumour and normal samples were obtained by the package through investigating the logarithmic-2-fold changes of the genes (logFC, the cut-off value of 0.5). The DESeq2 package also provides the Benjamini–Hochberg method to adjust *p*-value (padj). The significant level was set at padj < 0.05.

### 2.4. FANTOM-CAT Analysis

FANTOM- CAGE-Associated Transcriptome (FANTOM-CAT) is an efficient software to analyze lncRNAs structure and function (http://fantom.gsc.riken.jp/cat/ (accessed on 7 April 2021)). Zenbu tool was used to determine the type of sense–antisense overlap of the genes through FANTOM-CAT [27].

### 2.5. Real Time-Quantitative Polymerase Chain Reaction (RT-qPCR)

Total RNA was isolated from hFOB, SaOS-2, G-292, SJSA-1, HOS, 143B, U2-OS, MNNG/HOS and MG-63 using TRIzol reagent (Invitrogen Corp, Carlsbad, CA, USA). cDNA samples were generated using the Omniscript Reverse Transcriptase kit (Qiagen, Hilden, Germany) and Oligo dT15 primers (Promega, Madison, WI, USA) following the manufacturer’s protocols. RT-qPCR was conducted with SYBR Green master mix (Waltham, MA, USA). Primer sequences used for RT-qPCR were stated in Table 1.

Quantitative PCR was conducted using a Viia 7 Real-Time PCR machine (Applied Biosystems, Foster City, CA, USA). The thermal cycler protocol used for the RT-qPCR as following; 50 °C for 2 min, 95 °C for 10 min, followed by 40 cycles of 95 °C for 15 s, and 60 °C for 60 s. GAPDH was used as a housekeeping gene to normalize gene expressions. Relative gene expressions were calculated using the 2^−ΔΔCT^ method. Data analysis was performed using GraphPad Prism software, version 8 (GraphPad software, San Diego, CA, USA). The data are presented as the mean ± standard error (SE) of values from 3 independent experiments. Statistical significance was determined using one-way ANOVA, with *p* < 0.05 considered statistically significant.

## 3. Results

### 3.1. Participants’ Characteristics

The samples were collected after surgical removal of the affected bone from 21 Australian OS patients. Only 16 samples contained paired tumour and healthy bone biopsies. Total samples number were noted as 24 tumour and 16 healthy bone biopsies (Table 2).

### 3.2. Differential Gene Expression Analysis between Tumour and Normal Samples

**Figure 2:**
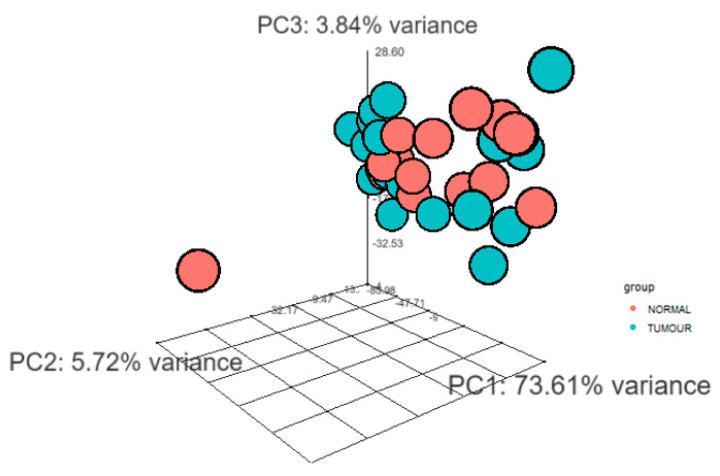
3D Principal component analysis (PCA) clustered transcript expression profiling of tumour and normal samples. 3D-PCA plot highlights the 3 principal components (PC1, PC2 and PC3). The groups have been marked by different colour; blue: tumour, orange: normal. The figure highlights that the tumour and normal samples were clustered separately.

Total RNA was collected from 24 tumour and 16 normal FFPE samples. Statistical data and differential gene expression analyses were performed through DESeq2 package for R. The 3D principal component analysis (PCA) plot has been provided insights into the similarities between tumour and normal samples and indicated the quality of the gene expression data (Figure 2).

Differential transcript expression levels between tumour and normal samples were obtained by the package through investigating the padj and logFC values. The genes without padj values (NA) and scientific names (NA) were excluded from the data.

**Figure 3:**
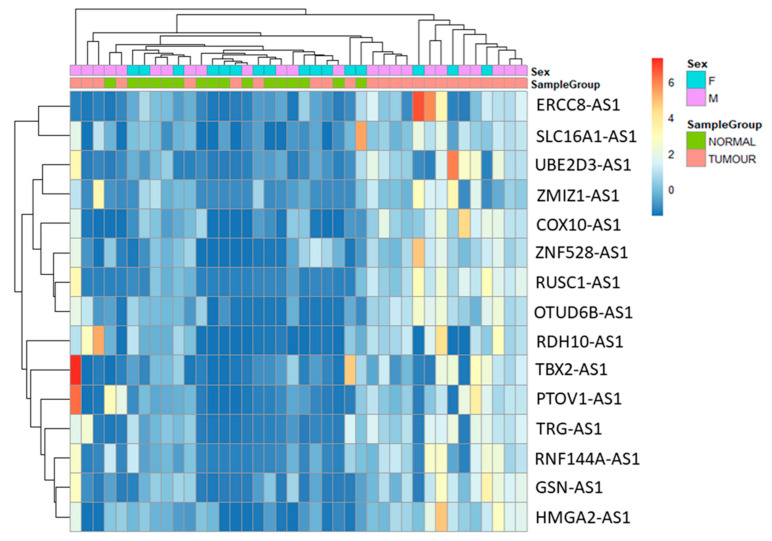
Heatmap of antisense lncRNAs expression comparison between the tumour (orange) and normal (green) samples.

**Figure 4:**
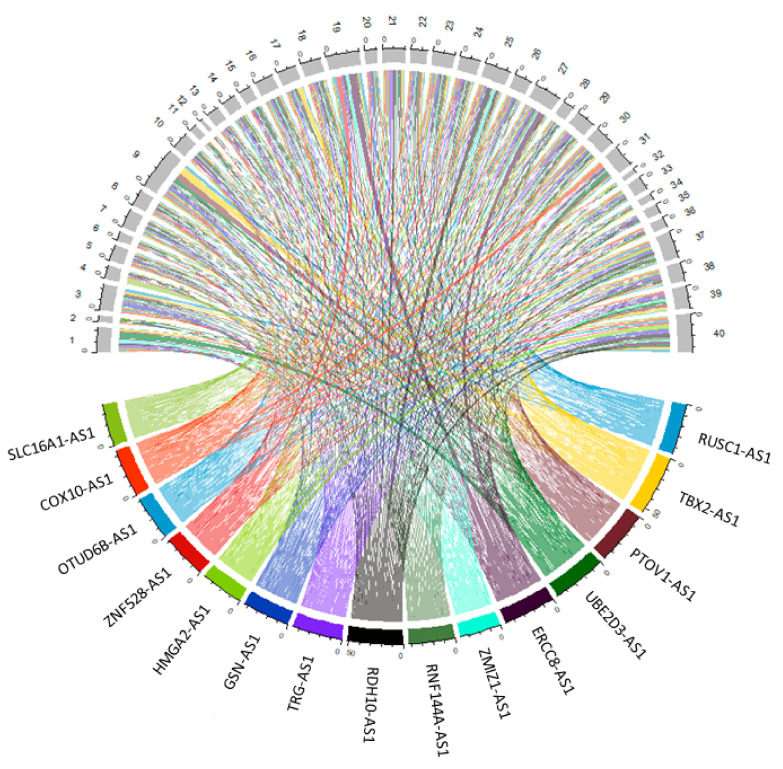
Circos plot visualizes the antisense lncRNAs expression in individual samples. 1, 3, 5, 7, 9, 11, 13, 15, 17, 19, 21, 23, 25, 27, 29, 31, 33, 34, 35, 36, 37, 38, 39, 40 are tumour samples, whereas 2, 4, 6, 8, 10, 12, 14, 16, 18, 20, 22, 24, 26, 28, 30, 32 are normal samples. Each colour represents a transcript.

The purpose of the study is to analyse antisense lncRNAs expression pattern between tumour and normal samples. The results showed that 15 antisense lncRNAs (RUN And SH3 Domain Containing 1-Antisense RNA 1 (*RUSC1-AS1*), T-Box Transcription Factor 2-Antisense RNA 1 (*TBX2-AS1*), Prostate Tumor-Overexpressed Gene 1 Protein-Antisense RNA 1 (*PTOV1-AS1*), Ubiquitin Conjugating Enzyme E2 D3-Antisense RNA 1 (*UBE2D3-AS1*), ERCC excision repair 8-Antisense RNA 1 (*ERCC8-AS1*), Zinc Finger MIZ-Type Containing 1-Antisense RNA 1 (*ZMIZ1-AS1*), Ring Finger Protein 144A-Antisense RNA 1 (*RNF144A-AS1*), Retinol dehydrogenase 10-Antisense RNA 1 (*RDH10-AS1*), T Cell Receptor γ Locus-Antisense RNA 1 (*TRG-AS1*), Gelsolin-Antisense RNA 1 (*GSN-AS1*), High Mobility Group AT-Hook 2-Antisense RNA 1 (*HMGA2-AS1*), Zinc Finger Protein 528-Antisense RNA 1 (*ZNF528-AS1*), OTU Deubiquitinase 6B-Antisense RNA 1 (*OTUD6B-AS1*), Cytochrome C Oxidase-Antisense RNA 1 (*COX10-AS1*) and Solute Carrier Family 16 Member 1-Antisense RNA 1 (*SLC16A1-AS1*) were upregulated in tumour samples compared to normal samples (Table 3). The data were visualized using heatmap (Figure 3) and circos plot (Figure 4). Further, the type of overlap of the antisense lncRNA transcripts was determined using FANTOM-CAT analysis and listed in Table 4.

### 3.3. Validation of Seven Novel Candidate Transcripts Expression Profiles through RT-qPCR

**Figure 5:**
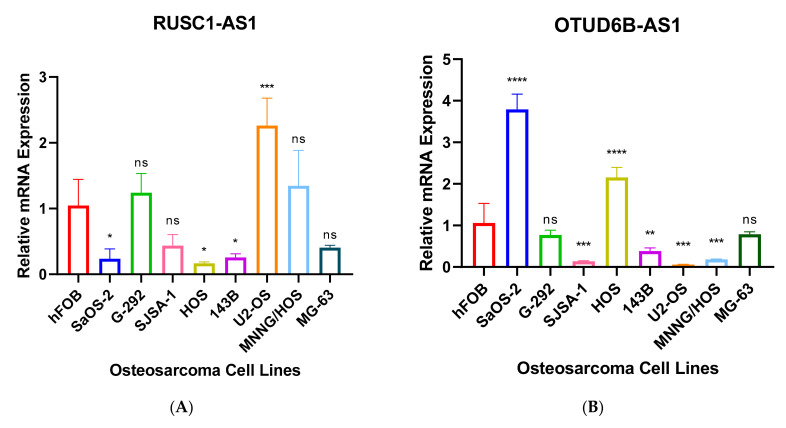

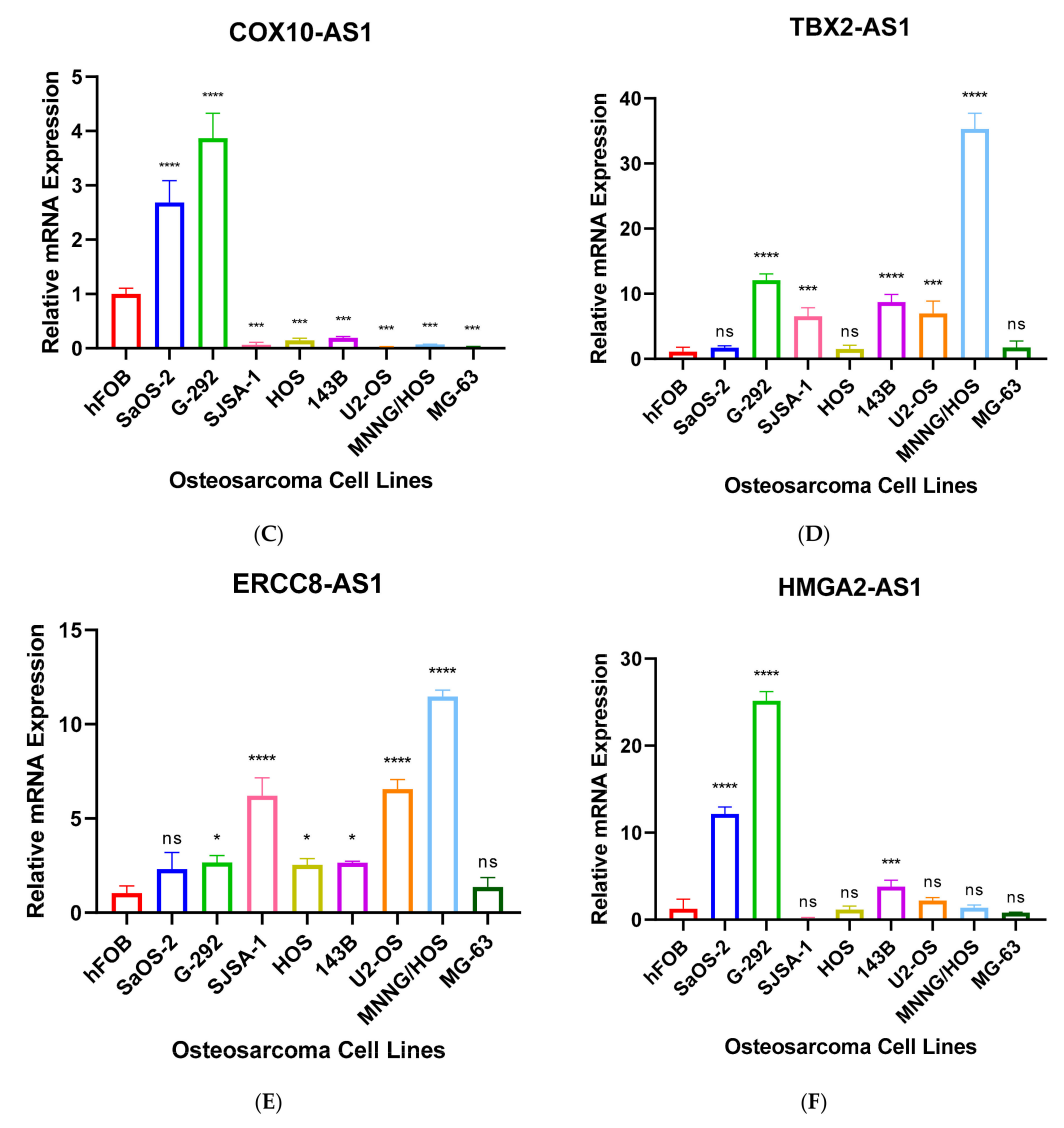

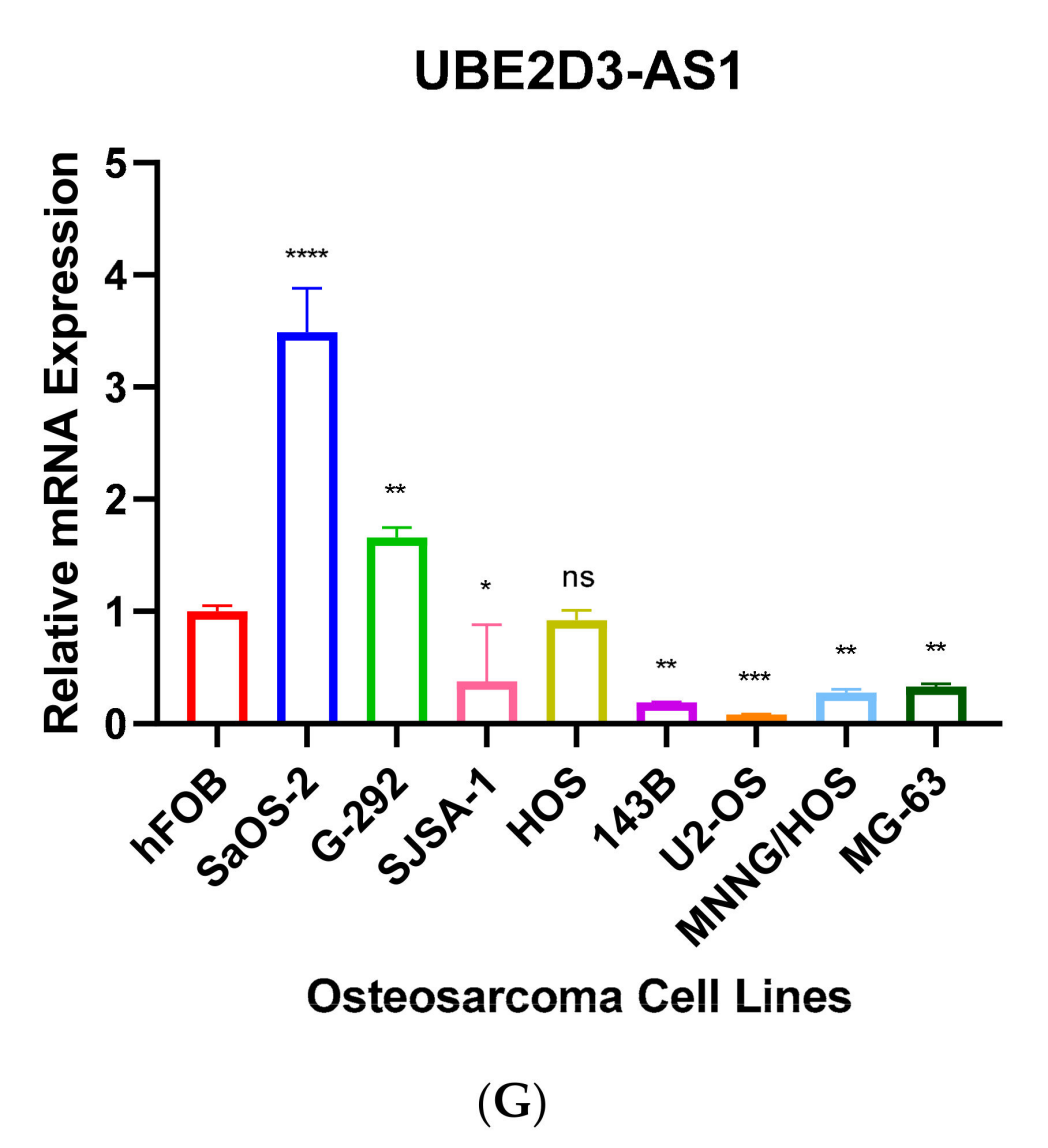
(**A**–**G**). RT-qPCR analysis of antisense lncRNAs. The transcripts expressions were determined using 8 different osteosarcoma cell line; SaOS-2, G-292, SJSA-1, HOS, 143B, U2-OS, MNNG/HOS, MG-63 and relative lncRNA expression obtained by hFOB expression. * *p* < 0.05, ** *p* < 0.01, *** *p* < 0.001, **** *p* < 0.0001.

Further, we performed RT-qPCR to validate RNA-seq results through 8 different OS cell lines, including SaOS-2, G-292, SJSA-1, HOS, 143B, U2-OS, MNNG/HOS and MG-63 and the expression values were compared to hFOB using the 2^−ΔΔCT^ method. The results have shown that only 7 antisense lncRNAs (*RUSC1-AS1*, *TBX2-AS1*, *UBE2D3-AS1*, *ERCC8-AS1*, *HMGA2-AS1*, *OTUD6B-AS1*, and *COX10-AS1*) have validation of transcript expression by RT-qPCR (Figure 5). Note that the RT-qPCR Ct values of remain 8 antisense lncRNAs were too high (>35 or not detectable) in hFOB cell line, suggesting the corresponding transcripts may not be expressed above the limit of detection of the RT-qPCR technique.

## 4. Discussion

NATs are a growing focus of cancer genomics studies. They have been dysregulated in various cancer types, are implicated in several malignant phenotypes [28,29,30], and are emerging as pre-dominant players in carcinogenesis through their involvement in gene expression regulation, epigenetic modification, evasion of growth suppressors and reprogramming energy metabolism [28].

In the present study, we analysed antisense lncRNAs expression patterns between tumour and normal samples. The RNA-seq result revealed that 15 antisense lncRNAs, *RUSC1-AS1*, *TBX2-AS1*, *PTOV1-AS1*, *UBE2D3-AS1*, *ERCC8-AS1*, *ZMIZ1-AS1*, *RNF144A-AS1*, *RDH10-AS1*, *TRG-AS1*, *GSN-AS1*, *HMGA2-AS1*, *ZNF528-AS1*, *OTUD6B-AS1*, *COX10-AS1* and *SLC16A1-AS1* were upregulated in tumour samples compared to controls. Further we performed RT-qPCR to validate the transcript expressions using OS cell lines and relative transcript expressions were obtained via osteoblast cell line-hFOB.

Dysregulation of *RUSC1-AS1* (also known as *C1orf104*) has been associated with several cancer types. According to some studies *RUSC1-AS1* is highly expressed in laryngeal squamous cell carcinoma, cervical cancer, and breast cancer cells [31,32,33]. Another study supports that the transcript promotes cell proliferation in hepatocellular carcinoma through modulating NOTCH signaling [34]. Our RNA-seq data also highlighted that the transcript was upregulated in OS samples compared to normal. According to our RT-qPCR result *RUSC1-AS1* was significantly upregulated in U2-OS cell line by more than a 2-fold (Figure 5B). Whereas, the transcript was dramatically downregulated in SaOS-2, HOS and 143B cell lines. The cell lines have different characteristics, morphology, and metastatic properties, consequently the differences can affect transcript expression. Further, passage number affects a cell line’s characteristics over time such as cell lines with high passage numbers can experience alterations in morphology, response to stimuli, cell growth rates, gene and protein expression and transfection efficiency, compared to lower passage cells [35,36,37,38,39].

A study highlighted a potential regulatory connection of *TBX2-AS1* and *TBX2*. The same study also suggested that *TBX2-AS1* tightly co-expressed with *TBX2* suggesting cis-regulation and their association with neuroblastoma [40]. Our RT-qPCR also has validated alongside with RNA-seq data that TBX2-AS1 was upregulated in G-292, SJSA-1, 143B, U2-OS, and MNNG/HOS by more than 15-fold compared to hFOB (Figure 5C).

The knockdown of heterogeneous nuclear ribonucleoprotein K (*hnRNPK*) eventually reduced *PTOV1-AS1* expression in HeLa cervical carcinoma cells. The study also investigated reduced expression of *hnRNPK* or *PTOV-AS-1* suppressed heme oxygenase-1 (HO-1) expression by increasing the enrichment of HO-1 mRNA in miR-1207-5p-mediated miRISC. The knockdown or decreased expression of either *hnRNPK* or *PTOV-AS-1* resulted in inhibition of the proliferation and clonogenic ability of HeLa cells [41]. We also observed upregulation of *PTOV-AS-1* in tumour samples compared to normal bone tissue. Interestingly, we also observed *hnRNPK* upregulation in tumour compared to normal samples (Figure S1).

The single-nucleotide polymorphisms (SNPs) of *ZMIZ1-AS1*, located at 10q22.3, has been associated with colorectal cancer and patients’ survival among Korean population [42,43]. The transcript also interferes with *ZMIZ1* gene regulating several tumour suppressors such as *SMAD4* and *p53* [44,45].

According to some studies *RNF144A-AS1*, also known as *GRASLND*, is highly expressed in bladder cancer, and overexpression of the transcript is correlated with poor prognosis. The same study also observed that the knockdown of *RNF144A-AS1* eventually inhibited cell proliferation, migration, and invasion in J82 and 5637 cell lines, in addition xenograft growth of cells was reduced compared to negative control in nude mice [46,47]. Another study highlighted *RNF144A-AS1* as an important regulator of mesenchymal stem cell chondrogenesis [48].

Over expression of *TRG-AS1* has been observed in several cancer types including squamous cell carcinoma of the tongue, hepatocellular carcinoma, and glioblastoma. Furthermore, *TRG-AS1* has been associated with poor prognosis [49,50,51].

Interestingly, *GSN-AS1* was downregulated in breast cancer patients [52]. The transcript was upregulated in OS tumour compared to normal samples in our study.

*HMGA2-AS1* positively regulates *HMGA2* expression and migration properties of *PANC1* cells through *HMGA2*. *HMGA2-AS1* is also correlated with poor prognosis in pancreatic cancer patients [15,53]. Not surprisingly, *HMGA2* plays a key role in cell proliferation and has been associated with various cancer types including colorectal, lung, gastric, colon, leiomyoma, and oesophageal squamous cell carcinoma [53,54,55,56,57,58,59,60]. The over expression of both *HMGA2-AS1* and *HMGA2* has been observed in our RNA-seq data (Table 3 and Figure S2). The RT-qPCR data also validated that *HMGA2-AS1* was significantly upregulated in SaOS-2, G-292 and 143B cell lines compared to hFOB (Figure 5F).

Interestingly, overexpression of *OTUD6B-AS1* inhibits cell proliferation, migration, invasion, and promotes cell apoptosis in colorectal cancer by sponging miR-21-5p and regulating *PNRC2* [61]. Another study has also suggested the similar findings, overexpression of the transcript inhibits cell proliferation, migration, and invasion by downregulation of microRNA-3171 [62]. Gang Wang et al. suggested that *OTUD6B-AS1* expression was downregulated in renal cell carcinoma via the Wnt/β-catenin signaling pathway and low expression of the transcript has been correlated with shorter overall survival than patients with high *OTUD6B-AS1* expression [63]. The overexpression of the transcript also reduced cell migration and invasion in thyroid carcinoma cells [64]. Whereas high expression of *OTUD6B-AS1* indicates poor prognosis in ovarian cancer [65]. The transcript is also upregulated in OS samples compared to normal bone tissues. The RT-qPCR result has also validated that the transcript was upregulated in SaOS-2, and HOS cell lines compared with hFOB (Figure 5A).

Chaoyang Zhou et al. found that *COX10-AS1* acts as a competing endogenous RNA to positively regulate *ACTG1* expression by sponging miR-361-5p and promotes glioblastoma cell proliferation and inhibits apoptosis [66]. The transcript is also upregulated in SaOS-2, and G-292 cell lines compared to hFOB by more than 2-fold in our RT-qPCR result (Figure 5D).

The upregulation of *SLC16A1-AS1* was observed in hepatocellular carcinoma, and glioblastoma [67,68]. Hong Yue Liu et al. suggested that the transcript was dramatically downregulated in non-small cell lung cancer and over expression of *SLC16A1-AS1* inhibits the cell viability and proliferation of lung cancer cell [69].

This is the first time of *ERCC8-AS1*, *UBE2D3-AS1*, *RDH10-AS1*, and *ZNF528-AS1* expressions in cancer have been reported. According to GeneCards (genecards.org (accessed on 10 April 2021)) ERCC8-AS1 has been associated with Cockayne Syndrome A and Cockayne Syndrome which one of main clinical features is cachectic dwarfism. It is a photosensitive, DNA repair disorder which has been associated with progeria that is caused by a defect in the transcription-coupled repair sub-pathway of nucleotide excision repair [70,71]. Another study has suggested that individuals with Cockayne Syndrome have mutations in *ERCC8* and *ERRC6*, resulting in defective transcription-coupled nucleotide excision repair. In addition, *ERCC1* or *ERCC4* mutation also have been reported in Cockayne Syndrome [72]. ERCC family also widely involved with Fanconi anemia which leads to bone marrow failure, several moderate skeletal abnormalities and a predisposition to leukemia and solid tumours [73,74]. Interestingly, downregulation of *ERCC4* has displayed worse survival outcome in OS patients [75]. Our RT-qPCR result also highlighted that *ERCC8-AS1* was upregulated in G-292, SJSA-1, HOS, 143B, US-OS and MNNG/HOS cell lines compared to hFOB (Figure 5E). *UBE2D3-AS1* is also upregulated in SaOS-2, and G-292 cell lines compared to hFOB (Figure 5G).

The limitation of this study is that some patients received chemotherapy may lead to gene expression alterations of the results. Another limitation of the study is the cell lines have had different passage numbers which may affect RT-qPCR results.

In conclusion, in this study, we performed RNA-seq analysis to identify differential expression of antisense lncRNAs between tumour and normal samples. The results highlighted that 15 antisense lncRNAs (*RUSC1-AS1*, *TBX2-AS1*, *PTOV1-AS1*, *UBE2D3-AS1*, *ERCC8-AS1*, *ZMIZ1-AS1*, *RNF144A-AS1*, *RDH10-AS1*, *TRG-AS1*, *GSN-AS1*, *HMGA2-AS1*, *ZNF528-AS1*, *OTUD6B-AS1*, *COX10-AS1* and *SLC16A1-AS1*) that were upregulated in tumour samples compared to normal. We also validated the transcript expression of *RUSC1-AS1*, *TBX2-AS1*, *UBE2D3-AS1*, *ERCC8-AS1*, *HMGA2-AS1*, *OTUD6B-AS1*, and *COX10-AS1* in OS cell lines compared to hFOB. The transcripts have not been sufficiently characterized and studied in cancer, especially in OS. A better understanding of the functions of antisense lncRNAs has the potential to elucidate the molecular pathogenesis of OS and other tumours, develop diagnostic and prognostic markers, and identify targets for novel precision therapies.

## Figures and Tables

**Table 1:** Human primer sequences used in RT-qPCR.

Transcript	Forward Primer	Reverse Primer
UBE2D3-AS1	TGAATGCTTATGCCGGTGGT	CGGCCCGAGCTA-GACTAAAG
OTUD6B-AS1	GACATATCCGGGTGACGTTTTT	TTGTTCCACTGTCTTCTGGCATT
COX10-AS1	TACCTCTGGGAAGTAC-GGGG	CACTTGCCACTGAAAGCACC
RUSC1-AS1	GAAAAGGATGGAGCAGCCGTCA	GGCTGAACGATGGAGACGAATG
HMGA2-AS1	GCAGCTTGTTTTCTGGGTGG	ACTTTGGGGGCAAAGTGTCA
ERCC8-AS1	GCCAAACCGAGATCACATGC	CACACAGTGGGAGCCTGAAT
TBX2-AS1	AACATCCAGGGCAATCTGGG	GTGCCGAGAGAATCGGTAGG
PTOV1-AS1	AGGCGATCCTCAGGAATGTG	AATAAGCAAGCCCCGGTTCA
RNF144A-AS1	CACACAGCAAGCTAGGA	ACTTTCCTTGCGAGGGTTGG
RDH10-AS1	TGACTACAGCGAGCAACAGC	TCCACTGAGACGGAAACTGC
TRG-AS1	CTCCTTCATTCCCTATTC	TTATGATGGCTACGATGT
ZMIZ1-AS1	TCTCAAGGCTCCGCTAGTCT	TCACCTGCATCCCCCAATTC
GSN-AS1	CCCATCAGCGGCTATCCAAA	TGGACATCGAGGAGGTCACT
ZNF528-AS1	ACACTGGCCTTAG-TCCTCCA	CTGCGCTTGTTTTCAGGGTT
SLC16A1-AS1	CCCTGGGAGGTAGGCCTTAT	TCTACCACCCTATGGGGCTC
GAPDH	GAAGGTGAAGGTCGGAGTC	GAAGATGGTGATGGGATTTC

**Table 2:** Characteristics of osteosarcoma patients who participated in this study.

Patient ID	Tumour	Normal	Gender	Age of Diagnosis	Site of Tumour	Chemotherapy	Vital Status
Q17B029593M	A7	A23	Female	26	Femur	Yes	Died from disease at the age of 26
Q17B045995J	A5	A29	Female	78	Femur	No	Died from disease at the age of 80
Q18B006524D	A28	A8	Female	74	Illium	No	Died from disease at the age of 74
Q18B009680H ’	A1	-	Male	17	Humerus	Yes	Died from disease at the age of 18
Q18B009680H ’	A12	-	Male	17	Humerus	Yes	-
Q18B014955A	A15	A23	Female	17	Femur	Yes	Died from disease at the age of 18
Q18B015603E *	A5	-	Male	26	Femur	Yes	Alive no evidence of disease
Q18B015603E *	A52	-	Male	26	Femur	Yes	-
Q18B018266Y	A1	E1	Male	58	T9-10 vertebra	Yes	Died from disease at the age of 58
Q18B028621H	A4	A1	Male	26	Femur	Yes	Alive no evidence of disease
Q18B034715Y	A6	A11	Female	19	Femur	Yes	Alive with metastatic disease
Q18B051017F	A1	A16	Male	69	Femur	Yes	Died from disease at the age of 71
Q19B001229R	A30	A22	Female	13	Femur	Yes	Alive no evidence of disease
Q19B005830Y	A2	-	Male	17	Tibia	Yes	Alive no evidence of disease
Q19B007088F	B10	B22	Female	17	Tibia	Yes	Alive no evidence of disease
Q19B013567K ^	A1	-	Male	63	Femur	No	Died from disease at the age of 64
Q19B013567K ^	A12	-	Male	63	Femur	No	-
Q19B021879L	A19	A21	Male	17	Tibia	Yes	Alive no evidence of disease
Q19B035672T	A1	-	Male	33	Femur	Yes	Alive no evidence of disease
Q19B051495P	B19	A2	Female	14	Humerus	Yes	Alive no evidence of disease
Q19B052024A	B2	B6	Male	36	Femur	No	Alive no evidence of disease
Q17B018941H	A12	B1	Male	36	Tibia	Yes	Died from disease at the age of 37
Q16B040208X	A33	A25	Male	17	Femur	Yes	Died from disease at the age of 18
Q15B001034Y	A15	B1	Male	19	Femur	Yes	Died from disease at the age of 20

’, *, ^ were from the same patients that the tumours were removed at different times. In the Tumour and Normal section of the table, A and B with the numbers represent participants’ sample code.

**Table 3:** The list of differentially expressed antisense lncRNAs transcripts with their corresponding log change values (log2FoldChange), *p*-value and padj (adjusted-*p*-value).

ENSEMBL	Symbol	Log2FC	*p*-Value	padj	Transcripts Name
ENSG00000225855.7	*RUSC1-AS1*	4.556139	3.83E-05	0.008835	RUSC1 antisense RNA 1
ENSG00000267280.5	*TBX2-AS1*	3.98406	0.001648	0.035197	TBX2 antisense RNA 1
ENSG00000268006.1	*PTOV1-AS1*	3.833228	0.000187	0.017564	PTOV1 antisense RNA 1
ENSG00000246560.2	*UBE2D3-AS1*	3.770542	0.003651	0.045301	UBE2D3 antisense RNA 1
ENSG00000233847.1	*ERCC8-AS1*	3.385903	0.004691	0.048827	ERCC8 antisense RNA 1
ENSG00000224596.8	*ZMIZ1-AS1*	3.366849	0.001591	0.035104	ZMIZ1 antisense RNA 1
ENSG00000228203.7	*RNF144A-AS1*	3.300567	0.002698	0.041347	RNF144A antisense RNA 1
ENSG00000250295.6	*RDH10-AS1*	3.227144	0.003145	0.043071	RDH10 antisense RNA 1
ENSG00000281103.2	*TRG-AS1*	3.160751	0.001404	0.033432	T cell receptor γ locus antisense RNA 1
ENSG00000235865.2	*GSN-AS1*	3.103048	0.002449	0.040134	GSN antisense RNA 1
ENSG00000197301.7	*HMGA2-AS1*	2.912661	0.000946	0.030737	HMGA2 antisense RNA 1
ENSG00000269834.6	*ZNF528-AS1*	2.774884	0.001737	0.03563	ZNF528 antisense RNA 1
ENSG00000253738.2	*OTUD6B-AS1*	2.764901	0.001943	0.03686	OTUD6B antisense RNA 1
ENSG00000236088.10	*COX10-AS1*	2.722786	0.004343	0.04782	COX10 antisense RNA 1
ENSG00000226419.8	*SLC16A1-AS1*	2.427554	0.001752	0.03577	SLC16A1 antisense RNA 1

**Table 4:** Antisense lncRNAs and their type of overlap with sense RNA.

Transcripts	Type of Overlap
*RUSC1-AS1*	Head-to-head
*TBX2-AS1*	Head-to-head
*PTOV1-AS1*	Head-to-head
*UBE2D3-AS1*	Embedded
*ERCC8-AS1*	Embedded
*ZMIZ1-AS1*	Head-to-head
*RNF144A-AS1*	Head-to-head
*RDH10-AS1*	Tail-to-tail
*TRG-AS1*	Head-to-head
*GSN-AS1*	Embedded
*HMGA2-AS1*	Embedded
*ZNF528-AS1*	Head-to-head
*OTUD6B-AS1*	Head-to-head
*COX10-AS1*	Head-to-head
*SLC16A1-AS1*	Head-to-head

## Data Availability

The data that support the findings of this study are available from the corresponding author upon reasonable request.

## Supplementary Materials

The following are available online at https://www.mdpi.com/article/10.3390/genes12081132/s1, Figure S1. Table of upregulation of *HNRNPK* in tumour samples (A) and boxplot of expression level in tumour and normal samples (B); Figure S2. Table of upregulation of *HNRNPK* in tumour samples (A) and boxplot of expression level in tumour and normal samples (B).

## References

[1] Wu C.C., Beird H.C., Andrew Livingston J., Advani S., Mitra A., Cao S., Reuben A., Ingram D., Wang W.L., Ju Z. (2020). Immuno-genomic landscape of osteosarcoma. Nat. Commun..

[2] Durfee R.A., Mohammed M., Luu H.H. (2016). Review of Osteosarcoma and Current Management. Rheumatology.

[3] Goryn T., Pienkowski A., Szostakowski B., Zdzienicki M., Lugowska I., Rutkowski P. (2019). Functional outcome of surgical treatment of adults with extremity osteosarcoma after megaprosthetic reconstruction-single-center experience. J. Orthop. Surg. Res..

[4] Lindsey B.A., Markel J.E., Kleinerman E.S. (2017). Osteosarcoma Overview. Rheumatology.

[5] Rothzerg E., Ingley E., Mullin B., Xue W., Wood D., Xu J. (2021). The Hippo in the room: Targeting the Hippo signalling pathway for osteosarcoma therapies. J. Cell Physiol..

[6] Lamplot J.D., Denduluri S., Qin J., Li R., Liu X., Zhang H., Chen X., Wang N., Pratt A., Shui W. (2013). The Current and Future Therapies for Human Osteosarcoma. Curr. Cancer Rev..

[7] Rothzerg E., Ho X.D., Xu J., Wood D., Martson A., Maasalu K., Koks S. (2020). Alternative splicing of leptin receptor overlapping transcript in osteosarcoma. Exp. Biol. Med..

[8] Igarashi K., Yamamoto N., Shirai T., Nishida H., Hayashi K., Tanzawa Y., Kimura H., Takeuchi A., Miwa S., Inatani H. (2014). Late recurrence of osteosarcoma: A report of two cases. J. Orthop. Surg..

[9] Ponting C.P., Oliver P.L., Reik W. (2009). Evolution and functions of long noncoding RNAs. Cell.

[10] Lekka E., Hall J. (2018). Noncoding RNAs in disease. FEBS Lett..

[11] Hangauer M.J., Vaughn I.W., McManus M.T. (2013). Pervasive transcription of the human genome produces thousands of previously unidentified long intergenic noncoding RNAs. PLoS Genet..

[12] Prensner R.J., Chinnaiyan A.M. (2011). The emergence of lncRNAs in cancer biology. Cancer Discov..

[13] Dey B.K., Mueller A.C., Dutta A. (2014). Long non-coding RNAs as emerging regulators of differentiation, development, and disease. Transcription.

[14] Geisler S., Coller J. (2013). RNA in unexpected places: Long non-coding RNA functions in diverse cellular contexts. Nat. Rev. Mol. Cell Biol..

[15] Ros G., Pegoraro S., De Angelis P., Sgarra R., Zucchelli S., Gustincich S., Manfioletti G. (2019). HMGA2 Antisense Long Non-coding RNAs as New Players in the Regulation of HMGA2 Expression and Pancreatic Cancer Promotion. Front. Oncol..

[16] Faghihi A.M., Wahlestedt C. (2009). Regulatory roles of natural antisense transcripts. Nat. Rev. Mol. Cell Biol..

[17] Liu J., Hu J., Corey D.R. (2012). Expanding the action of duplex RNAs into the nucleus: Redirecting alternative splicing. Nucleic Acids Res..

[18] Gallo A., Vukic D., Michalik D., O'Connell M.A., Keegan L.P. (2017). ADAR RNA editing in human disease; more to it than meets the I. Hum. Genet..

[19] Slotkin W., Nishikura K. (2013). Adenosine-to-inosine RNA editing and human disease. Genome Med..

[20] Britto-Kido Sde A., Ferreira Neto J.R., Pandolfi V., Marcelino-Guimaraes F.C., Nepomuceno A.L., Vilela Abdelnoor R., Benko-Iseppon A.M., Kido E.A. (2013). Natural antisense transcripts in plants: A review and identification in soybean infected with Phakopsora pachyrhizi SuperSAGE library. Sci. World J..

[21] Rosikiewicz W., Makalowska I. (2016). Biological functions of natural antisense transcripts. Acta Biochim. Pol..

[22] Li R., Sklutuis R., Groebner J.L., Romerio F. (2021). HIV-1 Natural Antisense Transcription and Its Role in Viral Persistence. Viruses.

[23] Modi A., Vai S., Caramelli D., Lari M. (2021). The Illumina Sequencing Protocol and the NovaSeq 6000 System. Methods Mol. Biol..

[24] Bolger A.M., Lohse M., Usadel B. (2014). Trimmomatic: A flexible trimmer for Illumina sequence data. Bioinformatics.

[25] Guo Y., Dai Y., Yu H., Zhao S., Samuels D.C., Shyr Y. (2017). Improvements and impacts of GRCh38 human reference on high throughput sequencing data analysis. Genomics.

[26] Love M.I., Huber W., Anders S. (2014). Moderated estimation of fold change and dispersion for RNA-seq data with DESeq2. Genome Biol..

[27] Severin J., Lizio M., Harshbarger J., Kawaji H., Daub C.O., Hayashizaki Y., Consortium F., Bertin N., Forrest A.R. (2014). Interactive visualization and analysis of large-scale sequencing datasets using ZENBU. Nat. Biotechnol..

[28] Zhao S., Zhang X., Chen S., Zhang S. (2020). Natural antisense transcripts in the biological hallmarks of cancer: Powerful regulators hidden in the dark. J. Exp. Clin. Cancer Res..

[29] Pelechano V., Steinmetz L.M. (2013). Gene regulation by antisense transcription. Nat. Rev. Genet..

[30] Balbin O.A., Malik R., Dhanasekaran S.M., Prensner J.R., Cao X., Wu Y.M., Robinson D., Wang R., Chen G., Beer D.G. (2015). The landscape of antisense gene expression in human cancers. Genome Res..

[31] Hui L., Wang J., Zhang J., Long J. (2019). lncRNA TMEM51-AS1 and RUSC1-AS1 function as ceRNAs for induction of laryngeal squamous cell carcinoma and prediction of prognosis. PeerJ.

[32] Guo Q., Zhang Q., Lu L., Xu Y. (2020). Long noncoding RNA RUSC1-AS1 promotes tumorigenesis in cervical cancer by acting as a competing endogenous RNA of microRNA-744 and consequently increasing Bcl-2 expression. Cell Cycle.

[33] Hu C.C., Liang Y.W., Hu J.L., Liu L.F., Liang J.W., Wang R. (2019). LncRNA RUSC1-AS1 promotes the proliferation of breast cancer cells by epigenetic silence of KLF2 and CDKN1A. Eur. Rev. Med. Pharm. Sci..

[34] Chen Y.A., Cheng L., Zhang Y., Peng L., Yang H.G. (2020). LncRNA RUSC1-AS1 promotes the proliferation of hepatocellular carcinoma cells through modulating NOTCH signaling. Neoplasma.

[35] Esquenet M., Swinnen J.V., Heyns W., Verhoeven G. (1997). LNCaP prostatic adenocarcinoma cells derived from low and high passage numbers display divergent responses not only to androgens but also to retinoids. J. Steroid. Biochem. Mol. Biol..

[36] Briske-Anderson M.J., Finley J.W., Newman S.M. (1997). The influence of culture time and passage number on the morphological and physiological development of Caco-2 cells. Proc. Soc. Exp. Biol. Med..

[37] Chang L., Woloschak G.E. (1997). Effect of passage number on cellular response to DNA-damaging agents: Cell survival and gene expression. Cancer Lett..

[38] Wenger S.L., Senft J.R., Sargent L.M., Bamezai R., Bairwa N., Grant S.G. (2004). Comparison of established cell lines at different passages by karyotype and comparative genomic hybridization. Biosci. Rep..

[39] Sambuy Y., De Angelis I., Ranaldi G., Scarino M.L., Stammati A., Zucco F. (2005). The Caco-2 cell line as a model of the intestinal barrier: Influence of cell and culture-related factors on Caco-2 cell functional characteristics. Cell Biol. Toxicol..

[40] Decaesteker B. (2018). TBX2 is a neuroblastoma core regulatory circuitry component enhancing MYCN/FOXM1 reactivation of DREAM targets. Nat. Commun..

[41] Shin C.H., Ryu S., Kim H.H. (2017). hnRNPK-regulated PTOV1-AS1 modulates heme oxygenase-1 expression via miR-1207-5p. BMB Rep..

[42] Song N., Kim K., Shin A., Park J.W., Chang H.J., Shi J., Cai Q., Kim D.Y., Zheng W., Oh J.H. (2018). Colorectal cancer susceptibility loci and influence on survival. Genes Chromosomes Cancer.

[43] Song N., Shin A., Park J.W., Kim J., Oh J.H. (2017). Common risk variants for colorectal cancer: An evaluation of associations with age at cancer onset. Sci. Rep..

[44] Alazzouzi H., Alhopuro P., Salovaara R., Sammalkorpi H., Jarvinen H., Mecklin J.P., Hemminki A., Schwartz S., Aaltonen L.A., Arango D. (2005). SMAD4 as a prognostic marker in colorectal cancer. Clin. Cancer Res..

[45] Rivlin N., Brosh R., Oren M., Rotter V. (2011). Mutations in the p53 Tumor Suppressor Gene: Important Milestones at the Various Steps of Tumorigenesis. Genes Cancer.

[46] Bi H., Shang Z., Jia C., Wu J., Cui B., Wang Q., Ou T. (2020). LncRNA RNF144A-AS1 Promotes Bladder Cancer Progression via RNF144A-AS1/miR-455-5p/SOX11 Axis. Oncol. Targets.

[47] Wang Y., Du L., Yang X., Li J., Li P., Zhao Y., Duan W., Chen Y., Wang Y., Mao H. (2020). A nomogram combining long non-coding RNA expression profiles and clinical factors predicts survival in patients with bladder cancer. Aging.

[48] Huynh N.P., Gloss C.C., Lorentz J., Tang R., Brunger J.M., McAlinden A., Zhang B., Guilak F. (2020). Long non-coding RNA GRASLND enhances chondrogenesis via suppression of the interferon type II signaling pathway. eLife.

[49] He S., Wang X., Zhang J., Zhou F., Li L., Han X. (2020). TRG-AS1 is a potent driver of oncogenicity of tongue squamous cell carcinoma through microRNA-543/Yes-associated protein 1 axis regulation. Cell Cycle.

[50] Sun X., Qian Y., Wang X., Cao R., Zhang J., Chen W., Fang M. (2020). LncRNA TRG-AS1 stimulates hepatocellular carcinoma progression by sponging miR-4500 to modulate BACH1. Cancer Cell Int..

[51] Xie H., Shi S., Chen Q., Chen Z. (2019). LncRNA TRG-AS1 promotes glioblastoma cell proliferation by competitively binding with miR-877-5p to regulate SUZ12 expression. Pathol. Res. Pr..

[52] Yang F., Lv S.X., Lv L., Liu Y.H., Dong S.Y., Yao Z.H., Dai X.X., Zhang X.H., Wang O.C. (2016). Identification of lncRNA *FAM83H-AS1* as a novel prognostic marker in luminal subtype breast cancer. Onco. Targets Ther..

[53] Jing Z., Guo S., Zhang P., Liang Z. (2020). LncRNA-Associated ceRNA Network Reveals Novel Potential Biomarkers of Laryngeal Squamous Cell Carcinoma. Technol. Cancer Res. Treat..

[54] Wu Y., Wang X., Xu F., Zhang L., Wang T., Fu X., Jin T., Zhang W., Ye L. (2020). The regulation of acetylation and stability of HMGA2 via the HBXIP-activated Akt-PCAF pathway in promotion of esophageal squamous cell carcinoma growth. Nucleic Acids Res..

[55] Zhang S., Mo Q., Wang X. (2019). Oncological role of HMGA2 (Review). Int. J. Oncol..

[56] Xi X., Teng M., Zhang L., Xia L., Chen J., Cui Z. (2020). MicroRNA-204-3p represses colon cancer cells proliferation, migration, and invasion by targeting HMGA2. J. Cell Physiol..

[57] Li Y., Qiang W., Griffin B.B., Gao T., Chakravarti D., Bulun S., Kim J.J., Wei J.J. (2020). HMGA2-mediated tumorigenesis through angiogenesis in leiomyoma. Fertil. Steril..

[58] Dong J., Wang R., Ren G., Li X., Wang J., Sun Y., Liang J., Nie Y., Wu K., Feng B. (2017). HMGA2-FOXL2 Axis Regulates Metastases and Epithelial-to-Mesenchymal Transition of Chemoresistant Gastric Cancer. Clin. Cancer Res..

[59] Gao X., Dai M., Li Q., Wang Z., Lu Y., Song Z. (2017). HMGA2 regulates lung cancer proliferation and metastasis. Thorac. Cancer.

[60] Wang X., Wang J., Wu J. (2021). Emerging roles for HMGA2 in colorectal cancer. Transl. Oncol..

[61] Cai Y., Li Y., Shi C., Zhang Z., Xu J., Sun B. (2021). LncRNA OTUD6B-AS1 inhibits many cellular processes in colorectal cancer by sponging miR-21-5p and regulating PNRC2. Hum. Exp. Toxicol..

[62] Wang W., Cheng X., Zhu J. (2021). Long non-coding RNA OTUD6B-AS1 overexpression inhibits the proliferation, invasion and migration of colorectal cancer cells via downregulation of microRNA-3171. Oncol. Lett..

[63] Wang G., Zhang Z.J., Jian W.G., Liu P.H., Xue W., Wang T.D., Meng Y.Y., Yuan C., Li H.M., Yu Y.P. (2019). Novel long noncoding RNA OTUD6B-AS1 indicates poor prognosis and inhibits clear cell renal cell carcinoma proliferation via the Wnt/beta-catenin signaling pathway. Mol. Cancer.

[64] Wang Z., Xia F., Feng T., Jiang B., Wang W., Li X. (2020). OTUD6B-AS1 Inhibits Viability, Migration, and Invasion of Thyroid Carcinoma by Targeting miR-183-5p and miR-21. Front. Endocrinol..

[65] Li N., Zhan X. (2019). Identification of clinical trait-related lncRNA and mRNA biomarkers with weighted gene co-expression network analysis as useful tool for personalized medicine in ovarian cancer. EPMA J..

[66] Zhou C., Jiang X., Liang A., Zhu R., Yang Y., Zhong L., Wan D. (2020). COX10-AS1 Facilitates Cell Proliferation and Inhibits Cell Apoptosis in Glioblastoma Cells at Post-Transcription Level. Neurochem. Res..

[67] Tian J., Hu D. (2021). LncRNA SLC16A1-AS1 is upregulated in hepatocellular carcinoma and predicts poor survival. Clin. Res. Hepatol. Gastroenterol..

[68] Long Y., Li H., Jin Z., Zhang X. (2021). LncRNA SLC16A1-AS1 is Upregulated in Glioblastoma and Promotes Cancer Cell Proliferation by Regulating miR-149 Methylation. Cancer Manag. Res..

[69] Liu H.Y., Lu S.R., Guo Z.H., Zhang Z.S., Ye X., Du Q., Li H., Wu Q., Yu B., Zhai Q. (2020). lncRNA SLC16A1-AS1 as a novel prognostic biomarker in non-small cell lung cancer. J. Investig. Med..

[70] Van der Pluijm I., Garinis G.A., Brandt R.M., Gorgels T.G., Wijnhoven S.W., Diderich K.E., de Wit J., Mitchell J.R., van Oostrom C., Beems R. (2007). Impaired genome maintenance suppresses the growth hormone--insulin-like growth factor 1 axis in mice with Cockayne syndrome. PLoS Biol..

[71] Karikkineth A.C., Scheibye-Knudsen M., Fivenson E., Croteau D.L., Bohr V.A. (2017). Cockayne syndrome: Clinical features, model systems and pathways. Ageing Res. Rev..

[72] Manandhar M., Boulware K.S., Wood R.D. (2015). The ERCC1 and ERCC4 (XPF) genes and gene products. Gene.

[73] Kashiyama K. (2013). Malfunction of nuclease ERCC1-XPF results in diverse clinical manifestations and causes Cockayne syndrome, xeroderma pigmentosum, and Fanconi anemia. Am. J. Hum. Genet..

[74] Rosenberg P.S., Greene M.H., Alter B.P. (2003). Cancer incidence in persons with Fanconi anemia. Blood.

[75] Rothzerg E., Xu J., Wood D., Koks S. (2021). 12 Survival-related differentially expressed genes based on the TARGET-osteosarcoma database. Exp. Biol. Med..

