# Supplementary material for: Upregulation of 15 Antisense Long Non-Coding RNAs in Osteosarcoma
Source: Genes (Basel). 2021 Jul 26;12(8):1132. doi: 10.3390/genes12081132 (PMC8394133; doi:10.3390/genes12081132)
Supplement: Supplementary file 1 [file genes-12-01132-s001.zip › supplementary.pdf]

Supplementary Results:

A)

ENSEMBL	Symbol	Log2FC	p-value	padj	Transcripts Name
ENSG00000165119.21	HNRNPK	2.850860127	0.003267926	0.043691026	Heterogeneous nuclear ribonucleoprotein K

B)

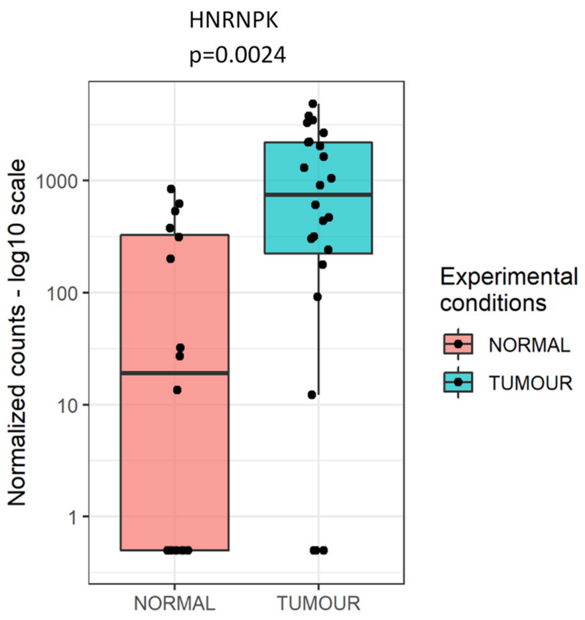

Figure S1. Table of upregulation of *HNRNPK* in tumour samples (**A**) and boxplot of expression level in tumour and normal samples (**B**). The Y-axis highlights normalized counts – log10 scale, and X-axis represents the sample type. P-value was calculated using Student's t-test, $p < 0.05$ considered statistically significant.

A)

ENSEMBL	Symbol	Log2FC	p-value	padj	Transcripts Name
ENSG00000149948.14	HMGA2	3.020716121	0.000255172	0.019611596	High-mobility group AT-hook 2

B)

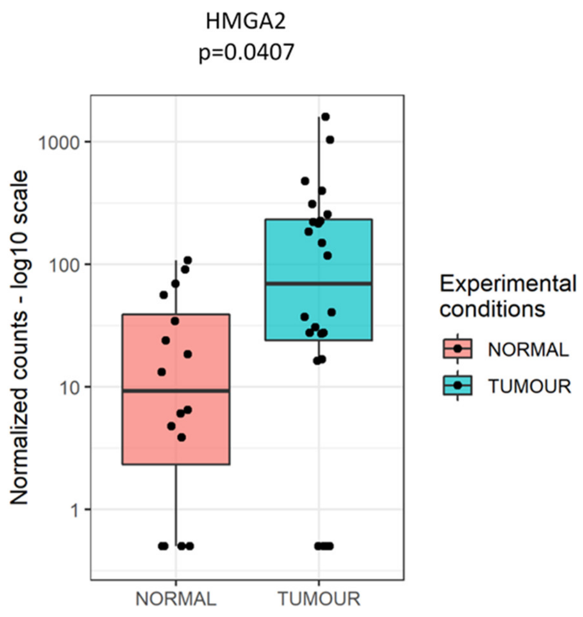

Figure S2. Table of upregulation of *HMGA2* in tumour samples (**A**) and boxplot of expression level in tumour and normal samples (**B**). The Y-axis highlights normalized counts – log10 scale, and X-axis represents the sample type. P-value was calculated using Student's t-test, $p < 0.05$ considered statistically significant.
